# A Critical Review of Bottom-Up Proteomics: The Good, the Bad, and the Future of This Field

**DOI:** 10.3390/proteomes8030014

**Published:** 2020-07-06

**Authors:** Emmalyn J. Dupree, Madhuri Jayathirtha, Hannah Yorkey, Marius Mihasan, Brindusa Alina Petre, Costel C. Darie

**Affiliations:** 1 Biochemistry & Proteomics Group, Department of Chemistry and Biomolecular Science, Clarkson University, 8 Clarkson Avenue, Potsdam, NY 13699-5810, USA; dupreeej@clarkson.edu (E.J.D.); jayathm@clarkson.edu (M.J.); yorkeyh@clarkson.edu (H.Y.); 2 Laboratory of Biochemistry & Molecular Biology, Faculty of Biology, “Alexandru Ioan Cuza” University of Iasi, 700506 Iasi, Romania; 3 Laboratory of Biochemistry, Department of Chemistry, “Alexandru Ioan Cuza” University of Iasi, 700506 Iasi, Romania; 4 Center for Fundamental Research and Experimental Development in Translation Medicine–TRANSCEND, Regional Institute of Oncology, 700506 Iasi, Romania

**Keywords:** mass spectrometry, proteomics, protein identification, protein characterization

## Abstract

Proteomics is the field of study that includes the analysis of proteins, from either a basic science prospective or a clinical one. Proteins can be investigated for their abundance, variety of proteoforms due to post-translational modifications (PTMs), and their stable or transient protein–protein interactions. This can be especially beneficial in the clinical setting when studying proteins involved in different diseases and conditions. Here, we aim to describe a bottom-up proteomics workflow from sample preparation to data analysis, including all of its benefits and pitfalls. We also describe potential improvements in this type of proteomics workflow for the future.

## 1. Proteomics

The term proteome refers to all proteins that are produced or modified by an organism (e.g., human [1], animal [2], plant [3], bacteria [4]) or living system (e.g., organ, cell culture, complex community from an environmental sample)). The term “proteome” and the first dedicated proteomics laboratory were introduced in 1994 by Wilkins et al. to describe proteins as a complement to genomic data [5]. However, the “whole” proteome of a particular cell, tissue, organ, or organism is yet to be identified. This is particularly difficult due to the vast variety of proteins and their isoproteins/proteoforms/protein species, which are expressed at different levels—from very abundant proteins, such as actin, to less abundant ones, such as transcription factors—in different cells, tissues, or organs. The variety of post-translational modifications (PTMs) in proteins, which may be stable or transient, is responsible for the vast number of proteoforms, which is an obstacle in most proteomics experiments. This, corroborated with the multiple limitations of proteomics methods, makes the quest to identify the proteome of any given organism a difficult one [6].

The proteomics field consists of a wide range of methodology, which has been largely driven by the modern development of involved technology. The concept of global protein analysis as a complete atlas of human proteins was proposed over 50 years ago [7]; however, proteomics research did not start until the mid-1990s. The beginning of proteomics research was sparked due to parallel developments in four areas: (i) two-dimensional gel electrophoresis (2D-PAGE) evolving into a robust method to rapidly separate proteins contained in biological complex mixtures [8]; (ii) the continuous development of mass spectrometry methods for accurate mass and chemical structure analysis [9,10]; (iii) the constant production of large-scale genome research and enormous number of peptide/protein sequences catalogued in several databases [11]; and (iv) the development of novel bioinformatics tools to simplify the analysis of large volumes of MS data aiming towards identifying intact proteins and their functional or pathological PTMs [12,13].

The field of proteomics comprises a bioanalytical discipline that performs large-scale studies on proteins [14] that may be rooted from a basic science perspective or a clinical one, i.e., proteins that are associated with a broad range of diseases and conditions by means of their altered expression levels and/or PTMs. In addition to fundamental protein research or development of proteomics-related instrumentation, the detailed analysis of the proteome for a specific type of cell (e.g., tumor, blood, or tissue) has the potential to permit the discovery of new protein biomarkers aimed towards diagnostic purposes and novel drug discovery [15,16]. Currently, the knowledge provided by proteomic research adds greatly to the genetic information generated from all genomics studies. The combination of proteomics and genomics research has the potential to play a major role in future biomedical studies and to impact the development of next generation diagnostic and therapeutic approaches.

Mass spectrometry (MS)-based proteomics has led to the possibility of characterizing and quantifying the protein profile of biological specimens, as well as the possibility to discover their complex interactions involved in various specific pathologies. For example, various proteomic approaches combined with genomic analysis have been used in cancer research for obtaining more information about the molecular basis of tumor genesis and the development of more effective anticancer therapies [17,18,19].

**Figure 1:**
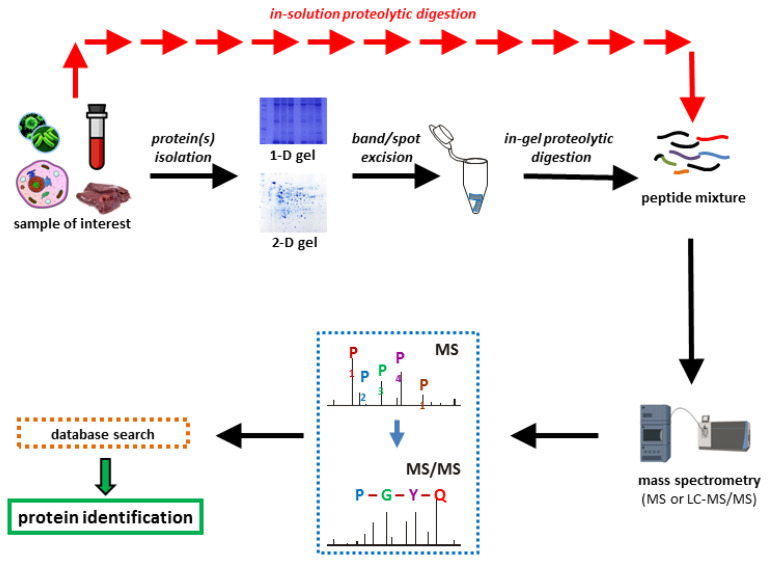
Workflow in a bottom-up proteomics experiment.

A typical bottom-up proteomics workflow (Figure 1) consists of several major steps: (i) isolation of the protein mixture from the studied biological sample, followed by (ii) quantification of isolated proteins concentrations (e.g., Bradford assay), then (iii) fractionation of proteins by gel electrophoresis or liquid chromatography methods. After fractionation, (iv) the proteins are proteolytically cleaved by enzymes (usually trypsin); followed by (v) a mass spectrometric measurement of the resulting peptides and (vi) a database search for protein identification.

The purpose of this review is to briefly introduce the bottom-up proteomic approach from a liquid chromatography–mass spectrometry perspective, to discuss its multidisciplinary development, its strengths and weaknesses, and to identify potential areas of improvement in the current methodology. These potential improvements will be relevant to the current problems in society, including biomedical, clinical, or environmental concerns.

## 2. Bottom-Up Proteomics

Most proteomic analyses use proteases to digest proteins into peptides with a predictable terminus. Peptides are then analyzed in an MS/MS instrument and their mass to charge ratio and predicted sequence is used to infer information about the proteins in the sample. All experimental set-ups starting from the analysis of peptides from complete protein digests and using a protein database to characterize the open-reading frame this peptide originated from, are grouped under the term “bottom-up” proteomics. 

Bottom-up proteomics utilizes the advantages that peptides have over proteins: peptides are more easily separated by reversed-phase liquid chromatography (RPLC), ionize well [20], and fragment in a more predictable manner. This translates into a robust methodology that enables high-throughput analysis, allowing for identification and quantification of thousands of proteins from complex lysates [21]. Today, bottom-up approaches using data-dependent acquisition (DDA) workflows are the core technologies in proteomics. Also termed shot-gun proteomics, these straightforward workflows generate large lists of protein identifications and were used for solving most of the available, complex, full proteomes available today, including the first drafts of the human proteome [22,23]. New data acquisition strategies such as selected reaction monitoring (SRM) further increase the quantification accuracy and reproducibility of bottom-up proteomics studies [24]. Data independent acquisition (DIA) is evolving to become a major driving methodology of the future. It attempts to combine a high number of protein identifications with consistency and accuracy in quantification of protein levels [25].

The hallmark of bottom-up proteomics is the extensive use of protease digestions, which has its drawbacks. Trypsin is the golden standard when it comes to shot-gun approaches and is being used for approximatively 96% of the deposited data sets in the Global Proteome Machine Database [26]. Trypsin is a very efficient protease with high catalytic activity that generates peptides with a basic arginine or lysine at the C-termini, which is ideal for collision-induced dissociation (CID) tandem mass spectrometry analysis [27]. Although efficient, when using trypsin, 56% of all generated peptides are ≤ 6 residues and thus too small to be identified by MS [28]. Moreover, only a fraction of these peptides yields useful fragmentation ladders [29]. The extensive usage of trypsin also leads to analytical tools that are optimized based on tryptic peptide properties. Furthermore, protein identifications are usually inferred by a limited number of typtic peptides. Hence, current bottom-up proteomics offers a rather biased and restricted coverage of the full proteome in a given sample, almost like “tunnel vision” of the proteome [28].

The limited sequence information inferred from small peptides is more often suffice to assign proteins clusters, and not always enough to identify proteoforms. Although possible through the measurement of mass shifts, protein isoform and PTM identification without prior knowledge is extremely limited in bottom-up proteomics.

## 3. Sample Preparation

As the proteomics field attempts to perform a comprehensive analysis of all the proteins in a given sample [30], sample preparation is the first and most important step in any experimental endeavor. If this step fails to perform, the sensitivity and throughput of the downstream steps are rendered pointless.

Genomics and transcriptomics, the other two major high-throughput -omics fields, benefit from the fact that nucleic acids are relatively uniform in terms of physiochemical properties. As such, “universal” standard methods and buffers are available that can be used to isolate nucleic acids of any size from almost any sample. No such thing exists in proteomics. The functional groups of the amino acid sidechains allow for such a great diversity in terms of protein charge and hydrophobicity, that a universal buffer will most probably never be available [31]. 

Nucleic acid-based technologies also benefit from the power of the PCR reaction, which allows the production of large quantities of target DNA in a single, reliable, and easy to do step; however, the proteomics field has nothing of the sort. Biological protein mixtures are characterized by a great diversity of proteins in chemistry, shape, and size, and, most importantly, by their range in abundance. Some proteins can be found in quantities as low as one protein per cell, while others up to several million per cell [32]. Despite the great selectivity and sensitivity of mass-spectrometers, no instrument available today can solve such complexity of range in a single measurement. Thereby, various fractionation strategies must be used in order to improve the depth and coverage of proteomic analysis [33].

### 3.1. The Good

Usually, methodologies for bottom-up proteomic sample preparation include the extraction of proteins from the biological matrix, removal of the nonprotein contaminants such as DNA, sugars, and lipids, removal of the residual salts that may form adducts during ionization, and protein fractionation to reduce sample complexity. 

Bottom-up proteomics utilizes unfolded proteins that allow for easy access for proteases since amino acids are more readily available, thus, generating more peptides for MS analysis. In this case, tissue or cell lysis is performed directly in a buffer containing strong denaturants (such as urea or guanidine) and ionic detergents (sodium dodecyl sulfate, SDS, or deoxycholate-SDC). In addition, nonionic zwitterionic detergents (such Triton X-100, NP-40, digitonin, or CHAPS) further help solubilize membrane proteins and can be used when less denaturing conditions are required [34]. Depending on the sample, sometimes protein depletion or enrichment is a crucial step—usually mandatory for serum or plasma samples where albumin represents more than 50% of the proteins. Several methodologies are available for removing high-abundance proteins [35,36] or for enriching cell surface proteins [37], phosphoproteins [38], or various enzyme subclasses [39,40,41]. The techniques that provide the highest selectivity and sensitivity for the low abundant proteins are affinity chromatography [42] and immunoprecipitation [43], but their applicability depends on the availability of affinity supports or antibodies, as well as the quality of said antibody for the target protein. 

For the removal of small molecules that may have originated from the biological sample or were added in the extraction/depletion/enrichment, many methods are available, including dialysis, buffer exchange, size exclusion, protein precipitation, chromatography, or electrophoresis [44,45]. Of all the desalting methods, precipitation with organic solvents (acetone or methanol/chloroform) is the least expensive, simplest, and most scalable option for desalting proteins prior to MS analysis [20]. 

Two forms of gel electrophoresis, SDS-PAGE and 2D-PAGE, have been extensively used in proteomics analysis as they solve two problems simultaneously—they remove salts, detergent, and other small molecules from the sample and they fractionate the proteins based on apparent molecular mass (SDS-PAGE) or charge and apparent molecular mass (2D-PAGE). SDS-PAGE has the advantage that it is inexpensive, straightforward, and highly reliable. Its separating resolution is higher than size exclusion chromatography, but not as high as 2D-PAGE. 2D-PAGE has a high resolution and, with the introduction of immobilized pH gradient (IPG) strips, its reproducibility has improved considerably, becoming the method of choice for most proteoform studies [46,47]. The development of PTM-specific stains for phosphoproteins (e.g., Pro-Q Diamond) and glycoproteins (e.g., Pro-Q Emerald, Dansylhydrazine) further increased the applicability of 2D-PAGE [48]. 2D gels are very useful for de novo sequencing of proteins from organisms with no genome sequences available and can complement the identification of protein isoforms and modified proteins [49].

### 3.2. The Bad

When preparing a sample for bottom-up proteomics analysis, many factors can cause the experiment to fail. Some examples include incomplete lysis, incomplete solubilization of the proteins, and contamination during sample handling with compounds that interfere with the downstream analysis steps, including MS. Some of the most troublesome compounds are detergents—both the detergents used for sample solubilization and those used for cleaning laboratory glassware. Detergent-contaminated samples and foul autosampler needles and LC piping reduce column capacity and performance and have poor ionization in both ESI and MALDI [20]. Triton X-100, Tween, or NP-40 contain polyethylene glycol (PEG) chains that elute throughout the LC and overwhelm the MS detector. SDS can cause complete signal suppression at levels as low as 0.01% [50]. Not all detergents are the same—some are considered safer for mass spectrometry, such as N-octyl-beta-glucoside and octylthioglucoside [51]. Moreover, several mass-spectrometry-compatible detergents are available, such as ProteaseMax (Promega), Rapigest (Waters), PPS Silent Surfactant (Expedeon), or Progenta (Protea), which degrade with heat and in the low pH of the acid containing LC-MS buffers [20]. Compounds leaching from poor quality plastics and fittings are also common contaminants. Phthalates, for example, are known to ionize well and overwhelm the mass spectrometer. An extensive list of contaminates in mass spectrometry is provided by Keller, 2008 [52].

In many proteomic experiments, the immunoaffinity methods are used to deplete the most abundant proteins. For example, there are many methods available that deplete albumin from human serum. Although these methods allow for the identification of a larger number of proteins due to the decrease in signal suppression, there may be problems with the nonselective loss of other proteins [53].

Keratins from skin and dust are another common contaminant, which takes up detector time, clutters the raw data, and reduces the number of useful spectra recorded. Keratins are commonly introduced when casting and handling gels and gel pieces [54]. It is thereby imperative to wear gloves and scrupulously wipe down surfaces to minimize this contamination. Other frequent proteinaceous contaminants are lysozyme, DNase, and RNase introduced in the cell lysis step, bovine serum albumin and other weight marker components from electrophoresis, as well as trypsin and other proteases from the digestion step [52]. A significant amount of MS instrument time is spent sequencing peptides from these abundant contaminant proteins and not peptides from the actual sample of interest [55]. Exclusion lists have been put together in an attempt to cleverly remove the burden of these contaminants [55]; however, these lists are useless in certain instances when, for example, keratins are expected to be present in the sample to be analyzed. In this case, it is difficult to distinguish between endogenous and contaminating keratins [56].

SDS-PAGE is an easy, inexpensive method to separate proteins prior to mass spectrometry analysis; however, its separating power is not very high. Although 2D PAGE has better separating power, it is still not uncommon to have multiple proteins in the same spot [57]. Along with this, proteins with a low copy number and hydrophobic proteins are difficult to detect in 2-D electrophoresis [58].

Ultimately, difficulties in sample preparation for bottom-up proteomics analysis are a result of the huge variability of protein properties. There is an overwhelming number of methods and technologies available, each tailored for specific samples and protein groups. With such variety to choose from, one will have a hard time deciding which method to pick and how they should adapt it to fit the specific sample. Moreover, most of the methodologies are long, multistep preparations that are prone to losses, biases, and contaminations, while being extremely time-consuming and labor-intensive. In the end, all of this ultimately contributes to poor reproducibility of the current sample preparation and fractionation methods [59]. 

### 3.3. The Future

Ideally, what is needed is a reliable and universal method to extract and fractionate proteins; one that is fast, does not require detergents, requires minimal steps and sample handling, and can be automated. This may be achievable with the development of new approaches that rethink sample preparation in bottom-up proteomics. Sample Preparation by Easy Extraction and Digestion (SPEED) is a new approach that does what its name suggests—reduces sample preparation to three easy steps: (1) acidification with pure trifluoroacetic acid (TFA) to lyse cells and extract proteins, (2) neutralization with TRIS base, and (3) digestion with proteases to generate peptides [59]. All steps and buffers required for sample preparation can be integrated for a straightforward and possibly automated sample preparation. Depending on the protein digestion mechanism, three types of integrated sample preparation methods are emerging: (1) in solution digestion, (2) immobilized-enzyme-reactor, and (3) on bed digestion methods [60]. Integrated in-solution digestion methods include filter-aided sample preparation (FASP) that repurposes centrifugal ultrafiltration concentrators in order to remove detergents, perform protein cleavage and isolate peptide fractions [61,62]. Derived from FASP, an encapsulated in-StageTip (iST) device has an enclosed tip chamber with an inserted membrane where lysis, denaturation, and alkylation take place [63]. The membrane serves both as a filter and as separation support. NanoPOTS are microfluidic chip devices that use nanodroplet-based processing methods. They allow in-solution digestion and processing of single cells and are reproducible [64]. Immobilized enzymatic reactor (IMER) methods make use of special columns that contain immobilized trypsin and can be directly coupled to LC-MS system for automated protein digestion and online LC-MS/MS analysis [65]. On-bead digestion methods perform reduction, alkylation, and digestion steps on functionalized beads by trapping proteins into a very limited void volume. Suspension trapping (STrap) is a method that uses tips packed with a quartz or glass filter and a hydrophobic C18 layer to perform sample clean-up and protein digestion in one step [66]. Single-Pot Solid-Phase-enhanced Sample Preparation (SP3) uses paramagnetic beads for the same purpose [67] and can be easily automated using a liquid handling robot [68]. These methods can be easily automated and integrated.

## 4. Conventional HPLC/Modern UHPLC Fractionation

Currently, liquid chromatography coupled with mass spectrometry (LC-MS) is a common and indispensable analytical technique for proteomic investigation. This coupling initiated the development of novel ionization methods and led to a broad range of interfaces aimed at separating various biological complex mixtures [69]. Atmospheric pressure ionization (API) was the first method to directly interface a solution stream with a mass analyzer [70]. Several thermospray units were also introduced as a breakthrough for modern LC-MS [71,72]. 

### 4.1. The Good

The main applications of HPLC in proteomics are rooted in the concept that peptides can be separated over a time and buffer gradient. This allows for maximum identification by the mass spectrometer, helps with the problem of ion suppression from coeluting peptides, and helps in the identification of the individual protein sequence. Significant research has been aimed towards developing HPLC methods to enable the resolution and identification of all generated peptides from digested proteins in a given proteome. This is a difficult task for many reasons. For example, a serum proteome may contain up to 20,000 proteins with a concentration dynamic range of 10^11^, which, by proteolytical digestion, may result in more than 600,000 peptides [73] excluding PTMs. Fractionation of such complex peptide mixtures is a critical aspect of their mass spectrometric identification. The general principle of HPLC fractionation of complex peptides mixtures is based on their interaction with a stationary phase (column) and a mobile phase (solvent gradient elution). The three major HPLC modes developed for peptide fractionation or sequential separation utilize differences in peptide size (Size-Exclusion Chromatography (SEC)), net charge (Ion-Exchange Chromatography (IEX)), or hydrophobicity (Reverse-Phase Chromatography (RP-HPLC)) [74,75]. 

The well-established proteomic methods approaching structural characterization of proteins (top-down and bottom-up approaches), have led to the use of HPLC methods in a particular mode [76]. For example, SEC [77] was used in top-down approaches to study intact proteins as a favored method for size-based separation. SEC has been mostly selected to analyze antibody–drug conjugates to determine their purity [78] and to purify recombinantly expressed proteins [79]. Since SEC is considered a low-resolution chromatographic method that requires diluted samples, the SEC technique was employed in a combination of columns with different pore sizes to achieve sufficiently high resolution in the separation of a complex protein mixture with a broad molecular weight range (10–223 kDa) [80].

Moreover, the combination of SEC with RP-HPLC, in a two dimensional (2D) separating platform exceeded a one-dimensional (1D) RP-HPLC experimental run with 4044 more unique proteoforms identified in a sarcomeric protein mixture [80]. This multidimensional HPLC was introduced as a shotgun approach to analyze very complex protein or peptide mixtures without performing gel electrophoresis, but achieving the same separating resolution as bidimensional gel electrophoresis (2D-PAGE) [81]. In the so-called multidimensional protein identification technology (MudPIT) [82,83], a protein mixture was subject to specific enzymatic digestion, usually using trypsin and endoproteinase LysC, and the resulting peptide mixture was separated by strong cation exchange (SCX) and reversed-phase high performance liquid chromatography (RP-HPLC) [84,85]. A combination of HPLC, liquid phase isoelectric focusing, and capillary electrophoresis was reported as a multimodular approach for obtaining a better separation of complex protein mixtures [86]. Recently, the large dynamic range and complexity of human cancer cells was overcome by employing a 2D separation using a high pH and low pH reversed-phase liquid chromatography technique, and 2778 proteoforms from 628 intact proteins were detected [87].

RP-HPLC is used in most bottom-up proteomic experiments for separating proteolytically generated peptides due to its high peak capacity, reproducibility, and robustness [88,89]. Both bottom-up and top-down proteomics approaches are fully dependent on employed separation technologies to: (i) provide large-scale proteome coverage in a given time; (ii) accomplish higher analytical throughput; and (iii) cover a broad dynamic protein concentration range, including trace amounts of distinct proteins. Many proteomic approaches use a IEX/RPLC [90,91] combination where octadecylsilanes (C18) remains the preferred RP ligand and the choice of ionic ligands experimentally depends on the class of peptides to be enriched and/or fractionated.

The development of an HPLC column with an “ideal and perfect” peptide/protein separation capability is an important, continuous research objective. The main types of analytical columns currently used in proteomics research present different characteristics with regard to the material composition and particle size packed into it, and the length and diameter [92]. Remarkable separation of complex peptide mixtures in proteomic studies was achieved using nano-LC/UPLC and capillary columns containing packed alkyl bonded C8 or C18 [93] and silica-based monolithic capillaries [94,95]. In proteomic analysis, the ability to handle very small amounts of biological material is crucial. Miniaturized HPLC separation systems have been developed by using fused silica capillary columns [96,97] or chip-based devices [98,99,100]. Monolithic capillary columns (and other columns, such as C8 or C18 columns) can be produced in the laboratory without the need of expensive media, packing solvents, and high-pressure packing instrumentation with the proper training [101]. Even if reproducibility issues of published protocols to produce such columns were reported, they remain cost-efficient and depend on the researcher’s capability to pack it perfectly.

Separation time is an important factor in nanoflow LC/tandem MS technologies, which has been shown by Mann et al. [102] where the results on the yeast proteome were compared using different mobile-phase gradient running times, identifying 5806 peptides in 140 min and 13,682 peptides in 480 min. Furthermore, improved fractionation in chromatography was shown as a straightforward approach to separate coeluting peptides and the pH of the mobile phase was shown to have an important impact on the retention, selectivity, and sensitivity of the separation [103].

In 1997, MacNair introduced a hybrid stationary phase for ultrahigh-pressure reversed-phase liquid chromatography (UHPLC) for rapid separation [104]. The difference between UHPLC and traditional HPLC is that UHPLC uses smaller diameter packing material in the column and higher pump pressures than HPLC. In UHPLC separation, the size of the particles is usually approximately 1.7 μm in diameter, versus the 3–5 μm diameter of HPLC column particles and requires a pressure of up to 1000 bar, versus the 50–600 bar range that is typical in HPLC instruments [105]. UHPLC has raised the level of performance of the separation with significant gain in resolution, speed, and sensitivity. The use of UHPLC doubled the peak capacity and increased the separation speed over nine-fold and the sensitivity by three- to five-fold, as compared with an HPLC run [106]. The problematic thermal effect occurring in the column by using high pressure in UHPLC system was overcome by their narrower diameters. However, as column diameters decrease, technical problems such as (i) sealing the LC system to resist leakage, (ii) very high backpressures at high flow rates, and (iii) contamination were reported. In terms of primordial advantages of UHPLC over HPLC, there is the faster speed and less solvent consumption, but its evident disadvantage is the price [107]. 

### 4.2. The Bad

A problem that remains in part unresolved by liquid chromatography is membrane protein solubility, which requires the use of special solubilization agents such as acidic solvents, detergents, and/or chaotropes that need to be diluted or even removed prior to proteolytical digestion or mass spectrometric analysis [108].

To optimize fractionation protocols using multidimensional separation, several factors need to be considered, including the impact of ionic strength, buffer capacity, pH response on the retention time behavior, and peak shape of proteins or peptides [109]. Moreover, when using multiple SCX /HPLC separation, the elution of the peptides from the SCX column is not precise and the same peptide will appear in several of the subsequent HPLC runs, reducing the amount of that peptide and thus decreasing the sensitivity. In samples that are too complex/concentrated, the first few buffer injections will contain the majority of singly charged peptides and di/triprotonated peptides are usually not trapped [110]. The major obstacle for liquid chromatography in using multiple parallel columns is adjusting the hardware and/or software to work in a synchronic manner. The price of the columns and their stability is not to be neglected. Future contributions in column technology require important developments so that LC is not restricted to the conventional single-column fractionation methods. 

Broadly, pharmaceutical and clinical laboratories seem to be willing to sacrifice resolution to gain analysis speed. This sacrifice may not be acceptable in fundamental research when analyzing complex peptide/protein mixtures to provide data for developing novel drugs or when identifying new biomarkers with diagnostic purposes. HPLC/UHPLC will continue to be a pivotal analytical technique that, in combination with high resolution mass spectrometry, will further raise the level of performance with significant increases in resolution, speed, and sensitivity required for elucidation of complex biological of proteomes [111]. 

### 4.3. The Future

HPLC/UHPLC-MS will remain very useful in “omics” sciences and reference methods will be developed, but the transition to various clinical applications is more desired. In recent years, immunoassays have been progressively replaced by HPLC-MS due to higher sensitivity, significantly less false positives, and the reduced costs of used reagents because multiple analytes can be measured simultaneously [112]. On the other hand, some drawbacks of HPLC-MS analysis include the high cost of instrumentation and the necessity for the initial method development and rigorous validation. As of today, universal chromatographic methods are not available, and each laboratory has to develop their own methods critically depending on their available infrastructure. The improvement of proteome coverage by first reducing sample complexity via chromatography will be a great challenge to researchers. One important requirement that gets increasingly supported by the literature is the fact that the current column hardware is no longer adequate to maintain the very high efficiencies and small peak volumes produced by the high-quality particles (continually smaller) and high-quality packing procedures. The proteomics field has also been utilizing very slow microflow rates in order to increase sensitivity, which cuts down on buffer costs, but also increases analysis time [113].

As several method parameters, such as pH, temperature, buffer concentration, and gradient time are varied simultaneously, a revolutionary software that predicts chromatograms based on HPLC method development is desired to determine the behavior of the separation. This is necessary in order to optimize complex sample separation and to economize resources spent developing and running wet lab activities. With the help of bioinformatics developments, in silico chromatography will be of great help in the near future to predict LC experimental flows for avoiding time, solvent, and sample consumption normally used in optimizing protocols for complex biological peptide/protein mixtures. Recently, a predictive algorithm of peptide and protein retention times in reversed-phase chromatography [114] was reported as a complementary experimental tool for proteomics.

## 5. MS Analysis: Instrumentation

MS-based proteomics is one of the chosen methods for complex protein sample analysis. It has established itself as a superior technology for complete characterization of proteins [29]. It is widely used for sequence analysis, protein–protein interactions, and identifying PTMs [29]. In general, a mass spectrometer consists of an ion source to ionize the analytes, a mass analyzer to measure the mass to charge ratio (m/z) of the analytes, and a detector that detects the number of ions at each m/z value. Electrospray ionization (ESI) is a commonly used technique to ionize peptides or proteins for MS analysis [115]. It ionizes a liquid solution of sample and hence can be coupled to liquid chromatography for separation [29,115]. There are four different type of mass analyzers used in proteomics: ion trap, quadrupole, time of flight (TOF), and Fourier transform ion cyclotron (FT-MS). These analyzers are the key to maintain sensitivity, mass accuracy, resolution, and to generate information rich ion mass spectra (MS/MS spectra) from peptide fragments [116]. They can be used individually in instruments or combined with each other to take advantage of the strengths of each [29,116]. 

It is also necessary for peptides be broken up further before being analyzed by the mass spectrometer, by a process known as dissociation. Different dissociation techniques exist for mass spectrometers, including (1) collision-induced dissociation (CID), (2) electron-capture dissociation (ECD), (3) electron-transfer dissociation (ETD), and (4) higher-energy collisional dissociation (HCD).

### 5.1. The Good

There is a diverse range of MS instruments that cover many possible applications in proteomics. Recent developments in the instrument design have led to new ion activation techniques as well as allowing for lower limits of detection. It has also increased the ability of tandem mass spectrometry for peptide and protein structure elucidation by improving the understanding of gas-phase ion chemistry [117]. In addition, the dynamic range of instruments allows one to optimize LC-MS and LC-MS/MS methods such as Data Dependent Analysis (DDA), Data Independent Analysis (DIA), Selected Reaction Monitoring (SRM), and Parallel Reaction Monitoring (PRM).

DDA is a common data acquisition strategy that selects the most abundant precursor ions for MS/MS analysis [118]. It takes the selection of peptide signals forward for fragmentation and matches them to a predefined database. The method allows for minimal selection of redundant peptide precursors [119]. The semirandom peptide sampling phenomenon in a DDA method has shown to increase the rates at which new peptides are identified, especially during replicate analysis. However, the reproducibility of the low abundant peptides between the runs, remain a challenge in the random sampling situation [118,120].

DIA is a method in which all the peptides within a defined m/z frame are subjected to fragmentation [121]. This method allows for accurate peptide quantification without being restricted to profiling only the predefined peptides of interest. In addition, DIA has the potential to overcome the random sampling problem and to reproduce and quantify low abundant peptides [121]. DIA offers several advantages over DDA for characterizing complex proteins. Unlike DDA, which sequentially detects, selects, and analyzes the individual ions, DIA systematically parallelizes the fragmentation of detectable ions within an m/z window, regardless of their intensity, thus providing a broader dynamic range of detected signals, improved reproducibility for identification, better accuracy and sensitivity for quantification, and enhanced protein coverage [122].

SRM is an MS-based technique for quantitative analyses. It is a nonscanning technique where selectivity is increased through fragmentation [123]. This method has the benefit of being able to control error rates in discovery proteomic experiments and can efficiently generate specific and quantitative assays for a large number of proteins and their PTMs by being both cost and time efficient [124]. It serves as a benchmark to all time segment methods [125]. PRM is another targeted method of quantitation performed using high-resolution mass spectrometers such as quadrupole-Orbitrap (q-OT). The development and application of higher-energy collisional dissociation (HCD) fragmentation enables MS/MS spectra to be acquired in the Orbitrap analyzer with high mass accuracy and high resolution. HCD is a beam-type collisional dissociation similar to the dissociation achieved in QQQ as well as QTOF mass spectrometers. An advantage of using q-OT is that both the discovery and targeted experiments can be performed on the same instrument, and it is convenient to transfer instrumental parameters such as collision energy, retention time, quadrupole isolation window, etc. [126]. This approach enables the acquisition of full MS/MS spectra of a targeted peptide with high mass accuracy and resolution, thus quantifying highly specific proteins. Similar to SRM, PRM can also validate the abundance of proteins and their PTMs [126]. While SRM and PRM are comparable, PRM is the most suitable for an attomole-level detection and quantification of multiple proteins in a complex sample [127]. Its simple and straight forward acquisition method in addition to its high selectivity and specificity of data acquired from high resolution and high mass accuracy makes it a powerful quantitation method [128]. Thus, one can choose from one of the previously mentioned methods based on their specific needs and optimize these methods in the instrument in order to acquire data in a short period of time with accurate peptide quantitation. 

Recently, the combination of the linear ion trap with the Orbitrap analyzer has benefitted the advances in high resolution MS greatly [129]. It can achieve high mass resolutions within a fraction of a second, which is important in both qualitative and quantitative applications. Moreover, it allows for the identification and quantification of a compound even in the presence of a background ion that has a nominally identical mass [129]. Complementing the ion trap and Orbitrap combination, the quadrupole mass filter was coupled to an Orbitrap analyzer known as the “Q Exactive” instrument, which features fast collision-induced dissociation peptide fragmentation with high energy because of parallel filling and detection modes, and features high ion currents because of an S-lens. This combination aids multiplexed operation at the MS and tandem MS levels, enabling joint analysis of HCD fragment ions in the Orbitrap analyzer and fragmentation of different precursor masses by the quadrupole analyzer, overall making Q Exactive an exciting instrument for proteomics [129]. SWATH-MS is a DIA–LC-MS technique that has become prominent for quantitative proteomics mainly due to its high quantitation accuracy, increased peptide coverage, generation of a digital map, and excellent reproducibility. In addition to that, it also permits qualitative analyses and allows for small molecule applications such as forensic analysis and the identification of metabolites and metabolomics [130]. MS instruments are usually compared to Nuclear Magnetic Resonance (NMR) instruments because they are expensive and require regular maintenance and troubleshooting. However, the robustness, the relative simplicity of sample preparation steps, high-throughput analysis, and sensitivity of MS remains unmatched [131,132]. 

Ion mobility mass spectrometry (IM-MS) uses electric fields to drag analytes through a buffer gas, separating the analytes whilst providing structural information, and has had many improvements in recent years [133]. The extra dimension that ion mobility provides increases the peak capacity in LC-MS workflows [134]. It is most common to couple a TOF mass analyzer with ion mobility; however, other analyzers can be used.

### 5.2. The Bad

Mass spectrometers are very expensive, delicate, and require a significant amount troubleshooting and maintenance. Although they are known to produce extensive information on proteins, the abundance of data can give rise to negatives, false positives, and unassigned spectra. There is a dynamic range of instruments to cover all possible applications; however, there is not one specific instrument that can perform all kinds of experiments [132]. MS detection oscillates with the concentration range of 10^4^ and 10^5^ and sometimes even 10^7^. Overall, the complexity, dynamic range of biological samples, and low abundance of disease-specific biomarkers remains a major challenge for proteomic biomarker discovery, and there is no MS instrument that can simultaneously address these challenges efficiently [135]. In addition, complete characterization of the proteome both quantitatively and qualitatively remains a challenge [136]. These problems can be remedied by reducing the sample complexity before introduction into the MS. Another major challenge is the analysis of a large number of samples in clinical studies or discovery proteomics where there is often experimental variability among the clinical samples. Additionally, the impact of single nucleotide polymorphisms (SNPs) on proteome analysis has still not been fully investigated [136]. Proteomics experiments for hundreds of samples are expensive and time consuming [132]. Very few proteomics techniques allow for high throughput analysis while simultaneously maintaining the sensitivity and robustness. DIA/SWATH, as mentioned above, is an alternative for proteomic analysis of clinical samples on a large scale [132,137]. 

### 5.3. The Future

Although mass spectrometers have the upper hand concerning their high throughput analysis and robustness, there is a need for MS with better sensitivity, resolution, and accuracy for analyzing samples such as proteins in the blood plasma. This will further allow the instrument to detect low-abundance proteins and their PTMs or interacting partners [132]. In addition, conventional MS has trouble resolving multiple charge states for species such as protein complexes of several hundred kilodaltons, especially for heterogenous samples such as heavily glycosylated proteins. Instruments such as charge detection mass spectrometry (CDMS), a variant of MS can help overcome such complexities by allowing the detection of m/z and charge states, making it easy to determine the mass of ions in a sample [138]. Advances in MS methods will aid in improving the chances of biomarker discovery for MS-based proteomics. Bottom-up proteomics is the workhorse for proteomic analysis. The middle and top-down approaches must advance in order to completely characterize the protein isoforms as well as the PTMs [136]. Overall, continued improvements are needed in both MS methods and technology in order to overcome the aforementioned challenges. 

## 6. Analysis of Mass Spectrometry Data

Analysis of MS data proves to be a limiting factor in many proteomics experiments. Although direct analysis of the raw data can be very useful, this practice can be tedious and time-consuming. There are many different software pipelines available for a more high-throughput and time-efficient approach; however, these programs have a great number of limitations. Although there are still many obstacles to overcome, in recent years, the limitations in proteomic data analysis have lessened, and high-throughput data analysis continues to improve tremendously.

**Figure 2:**
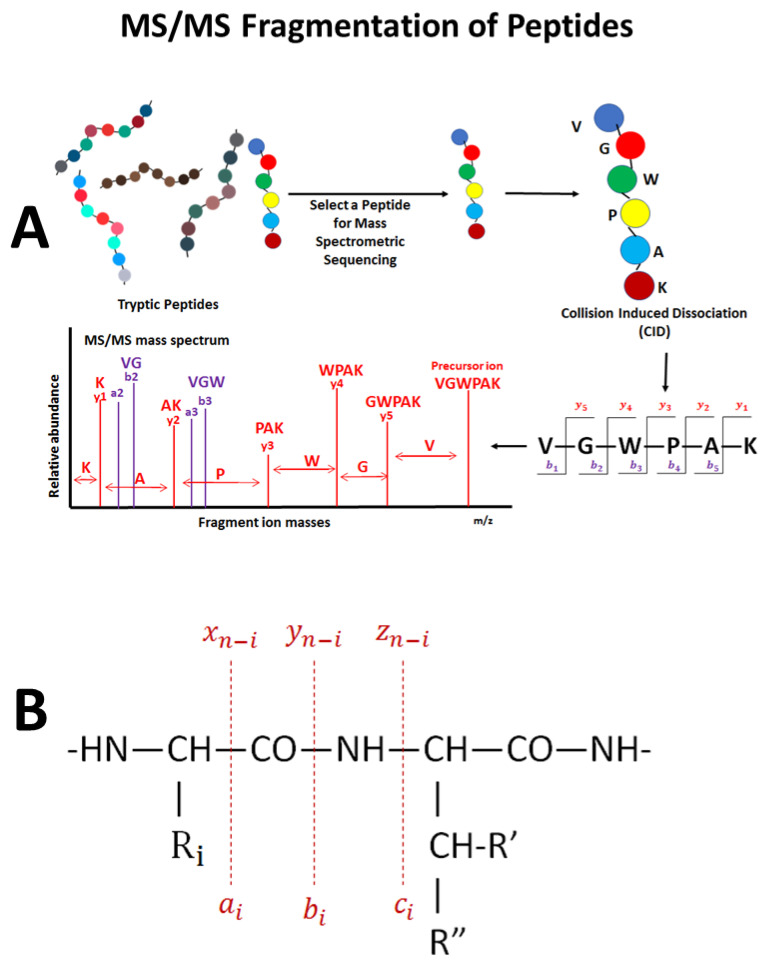
Correspondence between the peptide’s amino acid sequence and the fragment ion peaks that are produced in MS/MS (**A**) and the major theoretical fragment ions that are produced from fragmentation of a peptide (**B**).

It is important to understand the basics of peptide fragmentation before one can grasp a firm understanding of the current problems in proteomic data analysis, since most problems arise from lacking complete sequence information for many proteins. Peptides produced by enzymatic (i.e., trypsin) digestion will have between 5 and 20 amino acids. During collision-induced dissociation (CID) MS/MS fragmentation, the peptide is fragmented into many smaller fragment ions. In CID, these fragments are called y ions and are numbered from y1 (the C-terminal amino acid) to y(n), where (n) is the maximum number of amino acids in the peptide. A second type of ion in CID is the b ion. These ions start from the N-terminus, and end at the C terminus of the peptide. Both y and b ions are produced through fragmentation at the peptidic bond—the weakest bond in peptides (Figure 2). Additional ions are also produced in CID and observed in MS/MS: a and c (and b) ions from the N-terminus of the peptide and x and z (and y) ions from the C-terminus of the peptide (Figure 2). Predictability of peptide fragmentation (and production of the y, b, and a ions) allows for identification of peptide sequences using the de novo sequencing and in almost any database search algorithm. Different dissociation techniques produce different types of fragment ions. For example, ECD is complementary to CID as it provides more extensive sequence coverage. Disulfide bonds are preferentially cleaved in this method, and PTMs tend to stay intact; however, this method is specific to FTICR MS instruments [139]. Similarly, ETD fragments in the same type of way, creating longer c and z-type ions and preserving PTMs, but instead uses an RF quadrupole ion trapping device [140]. If used together, these dissociation techniques can give complementary information.

A particular advantage in proteomics-based analysis is that one protein upon enzymatic digestion can produce many peptides that can be useful in both identification and characterization of that protein. For example, if a protein can theoretically produce 50 peptides that can be analyzed by MS, only a few of those peptides are needed to identify that protein. These peptides can be native peptides and modified peptides (i.e., with Methionine oxidized), or can be native peptides with a different charge state. In addition, identification of these additional charge states for one peptide or unmodified and modified peptides can be particularly useful in characterization of that protein. For example, most therapeutic proteins have at least one or two methionines within their sequences. Knowing whether they are oxidized or not may be useful for long-term storage of those proteins since a methionine does not oxidize if it is buried inside the protein because it is not exposed to solvent. Having two or three charges may be useful in de novo sequencing, when the number of charges indicate the number of amino acids that can be protonated (i.e., only Arg, Lys, and His are/can be protonated). 

### 6.1. The Good

In a typical proteomics experiment, the raw data file is usually dependent on the MS instrument used and it should be first converted into a universal, readable file. More often, this file is a peak list and will concisely show the mass to charge ratio of precursor ions with their relative intensity and charge, as well as the mass to charge ratio of its fragment ions and their relative intensities. OmicX is an online a resource that lists different -omics software. Once the readable file is obtained, it can be submitted for a search against a target database containing theoretical peptide sequences. These sequences are generated by computationally performing theoretical digestions and MS/MS analysis of all the possible proteins/resulting peptides from a given genomic dataset. The search output is a list of peptide-spectrum matches (PSMs) that are used to identify individual peptides. The PSMs are then further used to infer the identity of proteins present in the sample [141]. Such searches can be performed through PLGS or through other programs such as Mascot (Matrix Science, London, UK), which has a subset of programs like the free Mascot Server, Mascot Daemon, Mascot Distiller, and Mascot Parser. This program can be used for protein identification and scoring. In this software, it is also possible to set the parameters to search for certain PTMs based on the mass shift. It is also possible to customize PTM searches to match specific experiments. In 1993, the Yates lab proposed a method that could be used to correlate mass spectral data with a predicted amino acid sequences in a protein database [142]. Following, a program known as Sequest came into the works [143]. PEAKS (Bioinformatics Solutions Inc, Waterloo, ON) is another protein identification software that also has a de novo sequencing function. Comet [144], is another search program which became publicly available in 2012. For experiments requiring relative quantification and statistical analysis, programs such as Scaffold (Proteome Software Inc., Portland, OR, USA) can be utilized. MaxQuant is an important quantitative software that is freely available [145].

Although typical database and spectral library searching are the main tools used for proteomic data analysis, in some instances, de novo sequencing of the raw MS/MS spectrum data is used to identify peptides in instances of novel proteins, mutations, and PTMs. This approach involves acquiring the data and determining mass and composition of peptides directly from the MS/MS spectrum and predicted fragmentation [146]. Current de novo sequencing algorithms include PEAKS [147], Lutefisk [148], PepNovo [149]. Mascot Distiller [150], Protein Prospector [151], Novor [152], UniNovo [153], and PeptideProphet [154]. 

Proteogenomics, first seen in 2004, in an approach that uses genomic and transcriptomic sequence information as a reference for MS/MS spectra to identify novel peptides [155]. Some examples of proteogenomic approaches include six-frame translation, ab initio gene prediction, and expressed sequence tags. The ability to pair genomic, transcriptomic, and proteomic methods has been the key factor in many discoveries [156,157,158,159].

The alignment of amino acids in proteins from different species can be a beneficial characteristic in proteomic analysis. If a well-developed protein database does not exist for a certain species, protein databases of similar species can be used. The protein hits that are obtained from these other databases can be used as a reference to determine what proteins are present in the species of interest; therefore, databases can be utilized that may not be specifically for the species of interest [160,161]. PEAKS software has a SPIDER algorithm that allows for cross-species homology search and detection of peptide mutations [147]. 

Many different approaches exist for quantitative proteomics experiments. In a simple, label-free method, a known concentration of an internal standard peptide can be spiked into samples prior to mass spectrometry analysis [148]. Likewise, an external standard peptide sample can be run in between samples on the mass spectrometer, and if the spectral count intensities of the standard runs are the same, the spectral counts/intensities of peptides in the actual samples can be compared as a relative quantitation [162,163,164,165]. In this case, the standard acts as proof that running conditions are identical enough that relative quantitation using spectral intensities can be used. There are also many available software for label-free quantitative proteomics, including MapQuant, MZmine, MsInspect, OpenMS, MSight, SuperHirn, and MaxLFQ [166,167,168,169,170,171,172]. The advantage of these types of analysis is that they are affordable and time-efficient. This type of quantitation works well enough for pilot and preliminary studies, but findings should be confirmed later using a more precise, reliable method. Labeling methods for quantification are also abundant, including methods such as isotope-coded affinity tag (ICAT), stable isotope labeling by amino acids in cell culture (SILAC), ^15^N/^14^N metabolic labeling, ^18^O/^16^O enzymatic labeling, isotope coded protein labeling (ICPL), tandem mass tags (TMT), and isobaric tags for relative and absolute quantification (iTRAQ) [173]. The absolute quantification (AQUA) method allows for the precise determination of protein expression and even post-translational modification levels by mimicking the exact peptide of interest, with the exception of stable isotope enrichment [174]. 

### 6.2. The Bad

The aforementioned type of proteomic data analysis is based off the premise that peptides are identified by matching their m/z information to a library of known proteins and their theoretical fragmentation. Similarly, peptides can be identified by matching MS/MS spectra against a library of theoretical spectra for that cell, tissue, organism, etc. which is a slowly growing practice. Therefore, it is assumed that all protein-coding gene sequences are known and well-annotated, and that all proteins encoded by these sequences exist in a database. Such databases include NCBI RefSeq or UniProtKB [175]. Unfortunately, this assumption is far from reality. The PTMs are not easily identifiable from genetic data; therefore, most modified peptides for the proteome of most organisms do not exist in a protein database, which makes proteomic data analysis a tricky task to tackle. As previously stated, de novo sequencing is a method that can be used in this instance. Although de novo sequencing is a very thorough type of analysis, problems with this technique include incomplete peptide fragmentation and low mass accuracy. These problems have been improved slightly in the most current software, but not totally. The downfall of proteogenomic approaches like six-frame translation, ab initio gene prediction, and expressed sequence tags is that they make very large reference databases that can sometimes be hard to work with [175].

Although the alignment of amino acids between different species can be beneficial, it can also be a complication in data analysis because most peptides are not unique to one specific protein or proteome. The NCBI Basic Local Alignment Search Tool (BLAST) shows regions of similarity between protein sequences [176]. Proteins of two different species can have more than 99% alignment of amino acids if they are closely related and can still have much more than 80% alignment if they are distantly related. The quality of alignment and conservation of sequences between proteins is interesting because it can hint towards species homology; however, it can be a complication when analyzing proteomics data.

When trying to perform quantitative proteomics experiments, the tools needed for a reliable analysis can be expensive. The cheapest way to quantify peptides is a label-free approach; however, variable running conditions for the mass spectrometer can lead to errors [177]. The amount of instrument time required for technical replicates is also a downfall when it comes to label-free approaches. In addition, it is not possible to accurately quantify the relative abundance of different peptides since these molecules have different physicochemical properties and thus will behave differently under mass spectrometry conditions [178]. The different labeling methods for quantitative proteomics also have their flaws. For example, ^15^N labeling relies on the number of nitrogen atoms present in the proteins, which is variable and can complicate analysis. A disadvantage of the SILAC method is that its cell culture origin limits its capabilities [178]. A universal problem with most labeling approaches is that there may be incomplete labeling of the peptides, which would cause errors in the quantitative analysis.

### 6.3. The Future

One of the major problems that needs to be addressed in proteomic data analysis is the lack of well-annotated protein databases. It is necessary for researchers to help develop these databases so that they can be used in future proteomics studies. When using a reference database for proteomic analyses, strict, relevant search parameters should be followed to decrease the likelihood of false positives. Whenever possible, smaller protein databases should be used instead of larger ones to minimize the false positives, peptide thresholds should be set to a reasonable tolerance, and relevant PTMs should be taken into consideration. In addition, if peptide identifications are particularly important, it is wise to verify them using the raw data spectrums.

## 7. False Positives, False Negatives, and Unassigned Spectra

The final and most biologically relevant result of a proteomics experiment is the identification of the proteins present in the sample. This is a two-step process that includes finding PSMs and inferring protein identity based on sequence alignments. Each step is dependent on a database, and, in each step, as the database grows larger, the probability to find peptides and hence proteins due to chance alone increases. Hits that are not actually present in the sample are called false positive identifications and used to be the most challenging problem in proteomics. 

### 7.1. The Good

In order to assess the quality of the PSMs and hence peptide identity, the false discovery rate (FDR) was introduced as the ratio between false PSMs and total number of PSMs. The lower the FDR, the more meaningful the results. Two major strategies for calculating FDR are available: target-decoy search strategy (TDS) [179] and mixture model-based methods [180]. 

In TDS, fictious decoy peptides are fabricated by reversing or shuffling the protein sequence from the target database. This decoy database is also used for searches, and the FDR level is controlled based on the amount of decoy peptides found in the search output [181]. This approach is the most widely used, although it has several disadvantages. First, as the target database grows larger, so does the decoy database, which increases search time and complexity. Some strategies for reducing [182] or eliminating the decoy database altogether [183] are available and have yet to find their way into the mainstream. Secondly, decoy database searches are more often biased by different peptide features or score functions [181], but some work has been done to overcome this issue [182].

When it comes to protein inference, many effective protein inference algorithms have been developed, such as ProteinProphet, ComByne, and MSBayesPro. For quality assessment of protein identification, two methodologies are available: *p*-value-based approaches that calculate a probability parameter for each identified protein, and FDR approaches that apply a single threshold to all proteins identified [141]. Previously, one of the major sources of false positive protein identification was identification of peptides based on a reduced number of fragment ions. However, this is no longer a problem, since identification of a protein based on one peptide alone is no longer acceptable within the scientific community. This strategy clearly eliminates true identification of some proteins, but it also eliminates any doubts in the proteomics dataset. 

The most commonly used computational strategy is to use database search algorithms based on searching acquired MS/MS spectra against a protein sequence database [184]. However, a large fraction of spectra remains unexplained despite a substantial improvement in the quality of MS/MS data acquired on modern mass spectrometers [184]. Novel sequences and the vast diversity of PTMSs remain unidentified in traditional database searches [185]. To develop a fast computational strategy that could be broadly applicable for searches using wide precursor mass tolerance of hundreds of Daltons (open database search), a novel fragment-ion indexing method was designed that provides orders of magnitude improvement in speed over existing tools [186]. This method implements a new database search tool called MS Fragger, which can perform open searches with variable modifications, thus identifying peptides with unknown alterations. It further allows open searches for data sets containing millions of MS/MS spectra and is applicable to data obtained from labeling-based qualitative proteomics experiments [186,187]. Open database searching offers a potential solution to the problem of inaccurate FDR estimates due to unaccounted peptide modifications in traditional narrow mass-window searches [186]. Using an independent platform tool like MS Fragger that is not limited to data from a particular MS instrument can be easily incorporated into most of the existing data analysis pipelines [186].

### 7.2. The Bad

A problem that will always persist in the proteomics community is false-negative identification of peptides, i.e., not detecting a peptide and a protein that contains it, for a variety of reasons. One such reason is physiological/pathological. For example, if a peptide should be identified in a sample, but it is physiologically/pathologically post-translationally modified (PTMs, i.e., phosphorylation, acetylation etc.), then the peptide is not found in any database search. While most researchers do search for common PTMs in peptides such as phosphorylation, they do not necessarily search for rare PTMs such as farnesylation, and are unable to search for unknown PTMs. 

While this problem could be solved (i.e., by expanding the database search using even more powerful software), the price is relatively high and each search will require a considerable amount of time. Another factor for false-negative identification of peptides is experimental. For example, if a peptide that should be identified is modified during the sample preparation process by artificial modifications/PTMs such as methionine oxidation, tryptophan oxidation, cysteine oxidation (to cysteic acid), iodoacetamide-based modification of cysteine (to carbamydomethyl cysteine) or cysteineless peptides, or acrylamide-based modification of cysteine-containing peptides (to propionamide), then these peptides will no longer be identified in a database search, unless all these artificial PTMs are selected in a database search. 

Physicochemical properties of proteins and peptides are also important and are not always considered. Hydrophobic proteins and natural PTMs in proteins are two examples of this. For example, hydrophobic peptides do not ionize well and, therefore, are not even found in the MS data. PTMs such as glycosylation and disulfide bridges can prevent trypsin cleavage and thus lead to false negative identification of peptides. An example of a peptide with a series of disulfide bridges, in which analysis of the precursor ions works well, but the disulfide bridges prevent a good fragmentation of these precursor ions, and thus leads to poor quality MS/MS spectra, was recently published from our lab [188]. 

Incomplete enzymatic digestions (usually trypsin) can lead to erroneous proteomics results and false negative identifications of some peptides. One such case is when one or more trypsin cleavage sites are missed. While 1–3 missed cleavages can easily be fixed (i.e., by database search with missed cleavages), peptides larger than 4–5 kDa that are produced by incomplete trypsin digestion are usually not identified, unless a specialized database is used. Furthermore, fragmentation of such large precursor ions does not produce good MS/MS spectra and therefore does not lead to the positive identification of any proteins [132].

Perhaps one of the biggest problems in a proteomics experiment is the unassigned MS/MS spectra (unpublished observations and [132]). Indeed, in a proteomics experiment, about 60–70% of the spectra are not assigned with confidence to any peptide (unpublished observations). This is true for experiments analyzing just one protein as well as analysis of a large set of proteins (unpublished observations). Again, multiple database searches that are sometimes customized can reduce the number of unassigned peptides to perhaps 50% or as low as 40% in the best-case scenarios, but there is still a lot of work that needs to be done in the area of bioinformatics and database searches. 

### 7.3. The Future

False positive results, false negative results, and unassigned spectra can be solved only by advances in database search and superior bioinformatics analysis of proteomes, i.e., additional software that allows identification of additional peptides that correspond to previously unassigned MS/MS spectra.

## 8. Conclusions

MS and proteomic methods have greatly advanced the field of protein analysis for both fundamental and clinical research. There are well-established methods for sample preparation and great instrumentation (both UPLC and MS) for MS analysis. There is also a diverse variety of database search engines. However, under the assumption that a proteomics experiment is done properly, the greatest challenge that we still face is a full, comprehensive database search of a proteomics dataset. It is clear that what proteomics needs most is a better, more powerful bioinformatics support for database searching.
